# Intracerebroventricular Administration of Amyloid β-protein Oligomers Selectively Increases Dorsal Hippocampal Dialysate Glutamate Levels in the Awake Rat

**DOI:** 10.3390/s8117428

**Published:** 2008-11-19

**Authors:** Sean D. O'Shea, Imelda M. Smith, Olive M. McCabe, Michelle M. Cronin, Dominic M. Walsh, William T. O'Connor

**Affiliations:** 1 Applied Neurotherapeutics Research Group, Conway Institute, University College Dublin, Dublin 4, Ireland; 2 Laboratory for Neurodegenerative Research, Conway Institute, University College Dublin, Dublin 4, Ireland; E-mail: dominic.walsh@ucd.ie; 3 Graduate Medical School, University of Limerick, Limerick, Ireland; E-mail: william.oconnor@ul.ie; 4 Materials and Surface Science Institute, University of Limerick, Limerick, Ireland

**Keywords:** Alzheimer's Disease, Amyloid *β*-protein, Microdialysis, Glutamate, GABA, Release

## Abstract

Extensive evidence supports an important role for soluble oligomers of the amyloid *β*-protein (A*β*) in Alzheimer's Disease pathogenesis. In the present study we combined intracerebroventricular *(icv)* injections with brain microdialysis technology in the fully conscious rat to assess the effects of *icv* administered SDS-stable low-n A*β* oligomers (principally dimers and trimers) on excitatory and inhibitory amino acid transmission in the ipsilateral dorsal hippocampus. Microdialysis was employed to assess the effect of *icv* administration of A*β* monomers and A*β* oligomers on dialysate glutamate, aspartate and GABA levels in the dorsal hippocampus. Administration of A*β* oligomers was associated with a +183% increase (p<0.0001 *vs.* A*β* monomer-injected control) in dorsal hippocampal glutamate levels which was still increasing at the end of the experiment (260 min), whereas aspartate and GABA levels were unaffected throughout. These findings demonstrate that *icv* administration and microdialysis technology can be successfully combined in the awake rat and suggests that altered dorsal hippocampal glutamate transmission may be a useful target for pharmacological intervention in Alzheimer's Disease.

## 1. Introduction

Extensive evidence supports an important role for soluble oligomers of the amyloid *β*-protein (A*β*) in Alzheimer's Disease (AD) pathogenesis [1]. To study the effects of soluble forms of A*β* we have taken advantage of an amyloid precursor protein-over-expressing cell line (referred to as 7PA2) that secrete A*β* peptides which migrate on SDS-PAGE at ∼4, 8 and 12 kDa and are recognized by antibodies specific for the mid-region, *N*- and *C*-termini of A*β*. These putative dimers and trimers potently block long-term potentiation (LTP) both *in vivo* and *in vitro* and perturb the memory of learned behavior, whereas A*β*monomer has no adverse effects [2–4]. The dorsal hippocampus (DH) is a key brain region implicated in learning acquisition and memory consolidation [5] and contains excitatory glutamate and aspartate-containing afferent and efferent pathways and inhibitory GABA interneurons. Evidence from both postmortem and *in vivo* studies suggests that the hippocampus is significantly involved in the pathophysiology of AD [6, 7]. Here we combine intracerebroventricular *(icv)* injections with brain microdialysis *via* a surgically implanted microdialysis probe in the dorsal hippocampus of the fully conscious Wistar rat to compare the effect of *icv* administration of A*β* oligomer with that observed for the A*β* monomer on dialysate glutamate, aspartate and GABA levels in the ipsilateral dorsal hippocampus.

## 2. Experimental Section

The experimental protocols employed in the project were approved by the University College Dublin, Animal Research Ethics Committee and the Department of Health and Children (Ireland) in accordance with the European Community Directive, 86/609/EC, licence number B100/3367. All experiments were carried out using the male Wistar rat supplied by Harlem U.K. Animals were housed individually in a thermoregulated environment (22°C) with a 12 hour light/dark cycle for the duration of the experiment. Food and water were available *ad libitum*.

### 2.1. Aβ Monomer/Oligomer Preparation

7PA2 conditioned medium (CM) was generated and fractionated as described previously [4]. Briefly, cells were allowed to condition glutamine- and serum-free Dulbecco's Modified Eagle's Medium and the resulting CM was concentrated 10-fold using a Centriprep Ultracel YM-3 filter. An aliquot of concentrate (1 mL) was chromatographed on a Superdex 75 10/300 GL column and eluted with 50 mM ammonium acetate pH 8.5 in 1 mL fractions. Aliquots of each fraction (300 μL) were used for Western blotting to identify monomer and oligomer-containing fractions (Figure 2A) and the remaining 700 μL of monomer and oligomer-enriched fractions were stored at −80°C pending use.

### 2.2. Microdialysis

**Figure 1.**
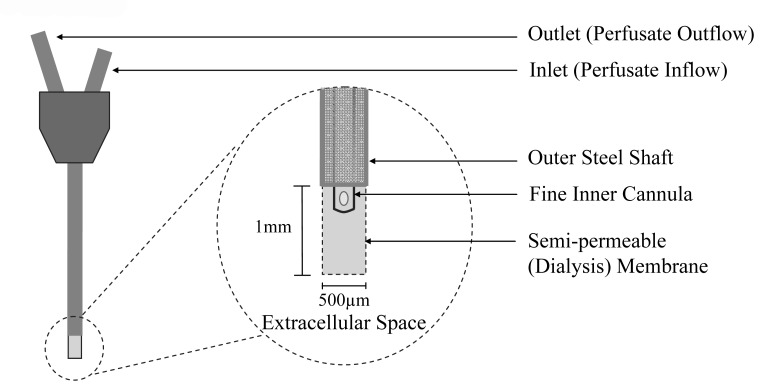
Schematic representation of the microdialysis probe employed in the present study showing the inlet cannula where perfusate enters the probe, the outlet where dialysate exits the probe and the semi-permeable dialysis membrane (1mm length and 500μm outer diameter) at the tip of the probe positioned in the extracellular space of the dorsal hippocampus.

Microdialysis enables the sampling of chemicals from the extracellular space of brain tissue via the microdialysis probe. The probe consists of a semi-permeable polycarbonate membrane (20,000 Dalton cut-off) mounted between the tip of an inner steel inlet cannula and an outer steel outlet shaft (Figure 1). Ringer perfusate is pumped at a controlled flow-rate into the membrane space of the probe through two holes in the inner cannula. Here, chemicals in the surrounding extracellular space passively diffuse across the membrane into the perfusate which then exits the probe for collection via the outer shaft. Thus, a representative proportion of the extracellular chemicals are measured by microdialysis as opposed to the absolute concentration of chemicals in the extracellular space.

### 2.3. Intra-Ventricular Cannulation and Intra-Hippocampal Microdialysis Probe Implantation

Each rat was anaesthetised under isoflurane (4–2% in air) inhalation using a Univentor 400 anaesthesia unit (Univentor, Malta) (delivered at 3.4 mL/min, air flow 500 mL/min) *via* a modified mouthpiece to maintain anaesthesia during surgery. The rat was then placed in a Kopf stereotaxic frame (David Kopf Instruments, USA) and stabilised with blunt ear bars to prevent damage to the tympanic membrane. A 1 mg/kg dose of rimadyl (Pfizer, U.K.) was administered (*s.c.*) as an analgesic. The incisor bar was set at -3.3 mm. During surgery the body temperature was continuously maintained at 37°C with a thermostatically regulated heating pad (CMA 150, Carnegie Medicin AB, Sweden). The parietal and the frontal bones were exposed by a sagittal incision of the scalp at the midline from the level of the eyes to the occipital protuberance. Two holes (0.8 mm outer diameter) were drilled according to stereotaxic co-ordinates [8] through the left side of the skull bone and the dura were carefully punctured. A 4 mm intraventricular *(icv)* injection cannula and a 1 mm concentric microdialysis probe (Carnegie Medicine AB, Stockholm, Sweden) were surgically implanted to the left lateral ventricle (AP -0.8 mm, ML +1.3 mm) and ipsilateral DH (AP -3.8 mm, ML -1.5 mm, DV -3.4 mm) respectively according to stereotaxic co-ordinates. A fresh injection cannula and microdialysis probe was used for each rat. Sterile Ringer solution (Baxter, U.K., composition in mmol/l concentrations: Na^+^ 147; K^+^ 4; Ca^2+^ 2.2; Cl− 156, pH ≈6) was perfused at a constant flow rate of 2 μL/min through the microdialysis probe using a microperfusion pump (CMA 100; Carnegie Medicin AB, Sweden) during probe implantation. Rats were given 48 hours to recover from surgery and received a high calorie (5% sucrose) solution to prevent dehydration and loss of body weight for the first 24 hours.

On completion of the experiment rats were sacrificed by pentobarbital (Euthatal, Rhone Merieux, Harlow, UK) overdose after which the brain was removed and immersion-fixed in 4% paraformaldheyde in preparation for visual verification of cannula and probe positioning. Animals with incorrect positioning were not included in the study.

### 2.4. Experimental Procedure

**Figure 2.**
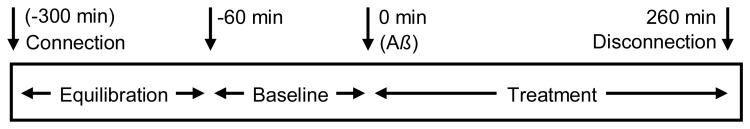
Schematic representation of the time course of microdialysis perfusion in the dorsal hippocampus and ipsilateral intracerebroventricular (*icv*) injection. Dialysate samples were not collected during equilibration (300 min) but were collected every 20 min thereafter yielding three baseline samples (−60 min) and 13 treatment samples collected 260 min following *icv* injection.

Each rat was placed in a hemispherical experimental microdialysis bowl (40cm diameter) (BAS, U.S.A.). The previously implanted microdialysis probe was connected to a microperfusion pump and ringer solution perfused at 2 μL/min. Following a 300 min washout period [9], three successive basal dialysate fractions (60 min) were collected and immediately following this a 20 μL aliquot of either monomer or oligomer was injected *icv* (Figure 2). The A*β* monomer-injected group acted as control. A further 13 successive fractions (260 min) were collected for GABA, glutamate and aspartate analysis.

### 2.5. Neurotransmitter Analysis

**Figure 3.**
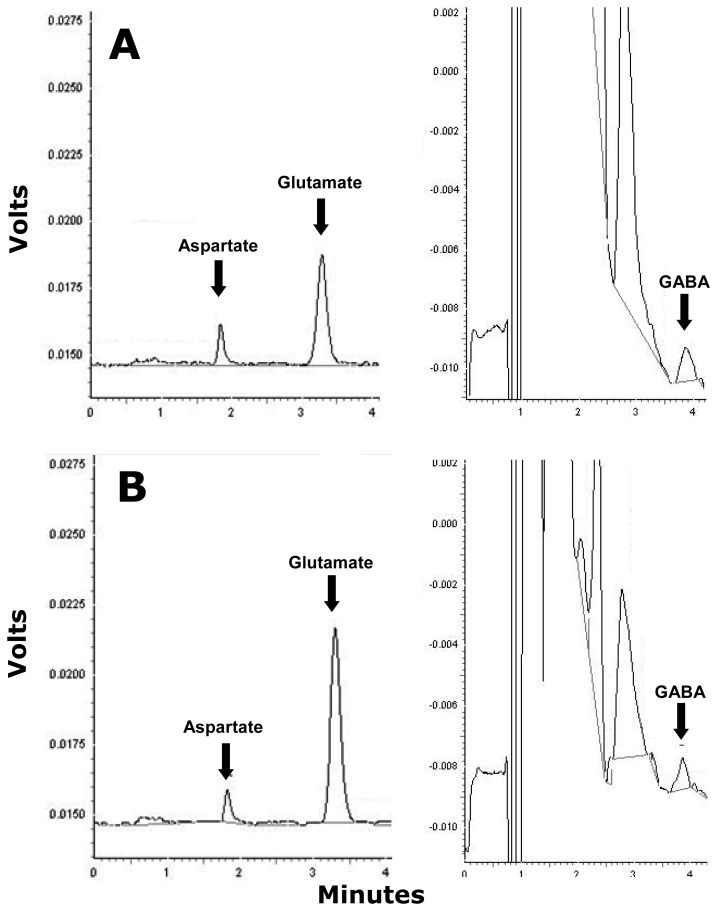
Sample high performance liquid chromatograms of glutamate and aspartate (left) and GABA (right) from 20 min dialysate samples collected from the dorsal hippocampus of the awake rat **(A)** 20 min prior to (basal) and **(B)** 200min following an ipsilateral *icv* injection of the A*β* oligomer.

Dialysate glutamate and aspartate levels were quantified using pre-column ortho-phthalaldehyde (OPA) derivatization in the microsampler followed by separation *via* isocratic High Performance Liquid Chromatography (HPLC) coupled to fluorimetric detection [10]. A 10 μL aliquot from each dialysate sample was pipetted into 0.3 mL eppendorfs and placed in a CMA 200 refrigerated microsampler (Carnegie Medicine AB, Sweden). Distilled H_2_O and a 2.5 μM glutamate/aspartate standard solution were analysed regularly to check for contamination and reliability. As part of the precolumn derivatization process the microsampler was programmed to add 5.4 μL of a derivatization reagent containing (OPA) and 2-mercaptoethanol to the dialysate sample prior to injection onto the HPLC column. The reaction was allowed to proceed for 120 seconds and then 14 μL of the derivatized sample was injected onto the column. The total run time sample separation and detection was typically 12 min. Retention time for glutamate and aspartate was typically 3.3 minutes and 1.8 minutes respectively with the limit of detection being 30 fmol/sample (Figure 3).

Dialysate GABA levels were detected following a pre-column OPA derivatization followed by reverse phase HPLC coupled to electrochemical detection [11]. A 10 μl aliquot from each dialysate sample was pipetted into 0.3 mL eppendorfs and placed in a CMA 200 refrigerated microsampler. Distilled H_2_O and a 25 nM GABA standard solution were analysed regularly to check for contamination and reliability. As part of the precolumn derivatization procedure the microsampler was programmed to add 0.5 μL of a derivatization reagent containing OPA and 2-methyl-2-propanethiol to the dialysate sample prior to injection onto the HPLC column. The reaction was allowed to proceed for 120 seconds and then 9 μL of the derivatized sample was injected onto the column. The total run time for sample separation and detection was typically 12–14 min. Retention time for GABA was 3.8 minutes and limit of detection was 50 fmol/sample (Figure 3).

### 2.6. Data Presentation and Statistical Analysis

Data are reported either as raw data or as normalised percentage change from basal levels (calculated as the mean of the two dialysate samples prior to *icv* injection) in the form of mean ± standard error of the mean (SEM). Statistical analysis between groups was performed using a two-way analysis of variance (ANOVA) with Bonferroni post-hoc analysis. P values are quoted where appropriate with P<0.05 considered significant.

## 3. Results and Discussion

### 3.1. Basal Dialysate Amino Acid Levels

Basal dialysate glutamate, GABA and aspartate levels (raw data) did not differ between the two experimental groups prior to ipsilateral *icv* A*β* monomer or oligomer injection (Table 1).

### 3.2. Effect of Aβ Monomer and Oligomer

**Figure 4.**
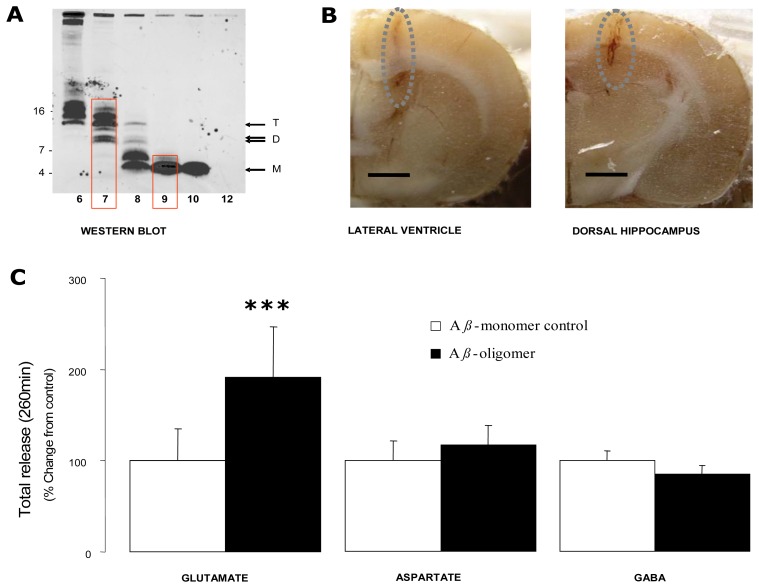
(A) Western blot analysis of size exclusion chromatography (SEC) fractionated 7PA2 conditioned medium (CM) with fractions (7 and 9) used for *icv* injection indicated by red boxes. The positions of A*β* monomer (M) (∼4 kDa), dimer (D) and trimer (T) bands (∼8, ∼12–14 kDa) are indicated with arrows. **(B)** Transverse sections of rat brain showing injection cannula track to the left lateral ventricle and microdialysis probe track in the ipsilateral dorsal hippocampus. Scale bar ≈ 2mm. **(C)** Dialysate glutamate, aspartate and GABA levels in the dorsal hippocampus following an intraventricular *(icv)* A*β* monomer and oligomer injection. Data are presented as histograms (mean ± SEM) representing percent change (260 min) from A*β* monomer-injected control group in the dorsal hippocampus following ipsilateral *icv* injection. See Table 2 and text for values. N=5-7 animals per group. ***=P<0.0001 *vs.* monomer-injected control (ANOVA).

An *icv* injection of the A*β* monomer had no effect on ipsilateral dorsal hippocampal dialysate glutamate, aspartate or GABA levels. In contrast, *icv* injection of the A*β* oligomer was associated with a gradual and prolonged selective +55% increase (*vs.* basal pre-treatment levels) in dialysate glutamate levels representing a +92% increase when normalised against the A*β* monomer-injected group (p<0.0001 *vs.* A*β* monomer-injected control) and was still increasing at the end of the experiment 260 min post-*icv* injection. Aspartate and GABA levels were not affected (Figure 4).

Dialysate levels of the excitatory amino acid neurotransmitters glutamate, aspartate and the inhibitory amino acid neurotransmitter GABA are at least in part derived from neuronal pools. Thus, dialysate glutamate and aspartate levels in the hippocampus are derived from local afferent and efferent pathways while GABA is derived from local interneurons. Falkenberg *et al.* [12] reported that injections of the glutamate receptor agonist quisqualate into the lateral entorhinal cortex increased dialysate GABA levels in the hippocampus CA1 region and that this GABA increase was attenuated by local administration of the sodium channel antagonist tetrodotoxin, indicating a neuronal origin. Furthermore, perfusion with the glutamate receptor agonist *N*-methyl-D-aspartic acid (NMDA) into the lateral entorhinal cortex increases dialysate glutamate release in the ipsilateral dorsal hippocampus [13]. In the present study the gradual and prolonged A*β* oligomer-induced increase in dialysate glutamate levels in the dorsal hippocampus suggests an increase in local afferent/efferent glutamate-mediated excitatory transmission, inhibition of reuptake and/or neurotoxicity while the lack of effect on aspartate indicates that glutamate and aspartate may originate from separate neuronal pools in this brain region. Previous studies report a differential release of aspartate and glutamate in the striatum, prefrontal cortex and hippocampus [14, 15]. The lack of an effect of A*β* oligomers on local GABA release demonstrates a neuronal specificity for local excitatory glutamatergic transmission within the dorsal hippocampus and suggests a neuronal specificity whereby release and/or reuptake from local glutamate-containing afferent and efferent pathways, but not local GABA interneurons, within the dorsal hippocampus are targets for A*β* oligomer action.

## 4. Conclusions

The present findings demonstrate that *icv* injection and microdialysis technologies can be successfully combined in the awake rat to demonstrate a selective and prolonged A*β* oligomer-induced increase in local glutamate levels in the ipsilateral dorsal hippocampus. The appropriate balance between the excitatory (glutamate) and inhibitory (GABA) neurotransmitter systems in this brain region is essential for normal neuronal function, while an imbalance may be neurotoxic [16]. Thus, the ability of low to sub-nanomolar concentrations of A*β* oligomers to induce this selective increase in excitatory glutamatergic transmission within the dorsal hippocampus suggests a highly sensitive neurochemical substrate for Alzheimer's Disease. Similarly, the lack of effect of similar concentrations of A*β* monomer is in keeping with our prior observations that monomer does not alter LTP or learned behavior, whereas oligomers potently inhibit LTP and perturb the memory of learned behavior. Taken together these findings could provide a useful avenue for pharmacological intervention in the treatment of Alzheimer's Disease.

## Figures and Tables

**Table 1.** Basal dialysate glutamate, aspartate and GABA levels (absolute levels) in dorsal hippocampus measured prior to ipsilateral *icv* injection of either the A*β* monomer or oligomer. Each value represents the mean ± standard error of the mean (SEM) of three consecutive 20 min dialysate fractions (60min) collected prior to ipsilateral *icv* A*β* injection (n= 5-7 animals per group).

	Glutamate (μM)	Aspartate (μM)	GABA (nM)
**A*β*-monomer**	1.07 ± 0.19	0.07 ± 0.01	6.22 ± 1.10
**A*β*-oligomer**	1.25 ± 0.52	0.05 ± 0.01	9.09 ± 2.41

**Table 2.** Dorsal hippocampal dialysate glutamate, aspartate and GABA levels following ipsilateral *icv* injection of either the A*β* monomer or oligomer expressed as percent change from basal (calculated as the mean of the two 20 min dialysate samples prior to *icv* A*β* injection). Each value represents the mean ± standard error of the mean (SEM) of thirteen 20 min dialysate fractions (260 min) collected following A*β* injection. See Table 1 for absolute basal levels. (n= 5-7 animals per group). ***=P<0.0001 *vs.* monomer-injected control (ANOVA).

	Glutamate	Aspartate	GABA
**A*β*-monomer**	− 19 ± 28	- 10 ± 20	+ 11 ± 11
**A*β*-oligomer**	+ 55 ± 45 ***	+ 6 ± 20	- 6 ± 11
